# Vitiligo: From mechanisms of disease to treatable pathways

**DOI:** 10.1002/ski2.460

**Published:** 2024-09-30

**Authors:** Gaurav N. Pathak, Isabella J. Tan, Ge Bai, Jimmy Dhillon, Babar K. Rao

**Affiliations:** ^1^ Department of Dermatology Rutgers Robert Wood Johnson Medical School Somerset New Jersey USA; ^2^ Department of Dermatology Rao Dermatology Atlantic Highlands New Jersey USA

## Abstract

Vitiligo is a chronic autoimmune‐mediated disease characterised by the loss of pigmentary melanocytes in the epidermis. Vitiligo is associated with loss of functional epithelium and significant reductions in quality of life with limited long‐term treatment options, highlighting a continued unmet clinical need. A comprehensive understanding of the pathophysiology and newly investigated treatment pathways may guide multimodal treatment strategies and identify future drug targets. The pathology of vitiligo is multifactorial; however, environmental insults in genetically susceptible populations may lead to disease development. Autoreactive CD8+ T‐cells that target melanocytes and release inflammatory mediators, including interferon‐γ and interleukins 2, 6, 15, 17 and 33 among others, have been identified in vitiligo pathogenesis. Treatment modalities for vitiligo revolve around six broad disease concepts, including procedural modalities (tissue and cellular grafting), phototherapy, stem cells, anti‐inflammatories, genetic polymorphisms and antioxidants/vitamins/herbals. Genetic polymorphisms, such as catalase gene variations and toll‐like receptor polymorphisms, along with stem cell targets such as melanocytes derived from stem cells, have been implicated in vitiligo onset and possible treatment. Novel JAK‐STAT inhibitors have been recently investigated for vitiligo, whereas topical corticosteroids and calcineurin inhibitors continue to be used. Vitamin D, vitamin E, zinc, copper, piperine, pseudo catalase and other vitamins/herbals may improve vitiligo outcomes primarily through antioxidant supplementation pathways. Future studies should investigate alternative drug pathways and targets implicated in vitiligo in large patient cohorts, as well as treatments that target suspected causative immune cells, including memory T‐cells, which may provide long‐lasting disease‐free remission.

## Graphical abstract

Vitiligo is a chronic autoimmune‐mediated disease characterised by the loss of pigmentary cells in the epidermis associated with comorbidity and reductions in quality of life. The pathology of vitiligo is theorized to be multimodal including auto‐reactive CD8+ T‐cells and inflammatory mediators including IFN‐γ and interleukins 2, 6, 15, 17, and 33. Novel treatment options are aimed at restoring pigmentation through procedures (grafting), phototherapy, stem cells, anti‐inflammatories and antioxidants.

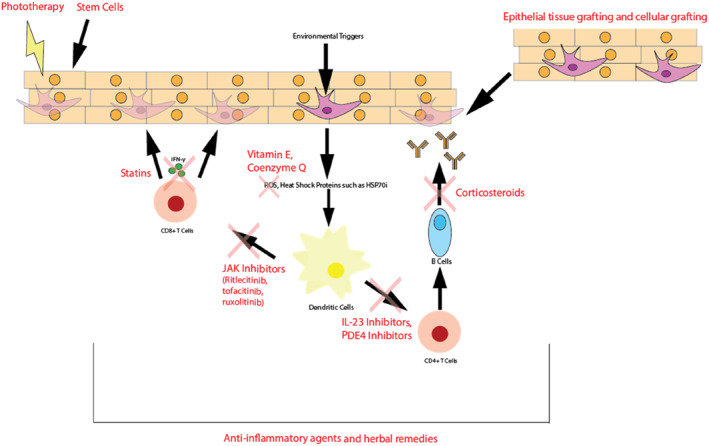

**What is already known?**

- Vitiligo is an autoimmune‐mediated disease characterised by loss of functional epithelium associated with limited long‐term treatment options and significant reductions in quality of life.
- Autoreactive CD8+ T‐cells, memory T‐cells, interferon‐γ and interleukins have been implicated in vitiligo pathophysiology.

**What does this study add?**

- Corticosteroids, calcineurin inhibitors and novel JAK‐STAT inhibitors, such as ruxolitinib, remain the mainstay of treatment. Vitamin D, vitamin E, zinc, copper, piperine and other herbal medications may improve disease severity.

## 1 INTRODUCTION

Vitiligo is a chronic autoimmune disease characterised by the loss of pigmentary melanocytes, resulting in white macules and patches on the skin commonly affecting the hands, face and feet. Vitiligo has a 0.5%–2% global prevalence, including nearly 1.9–2.8 million adults in the US (approximately 0.75%–1.11% overall prevalence).^1^ Alarmingly, about 40% of adult vitiligo cases may be undiagnosed, highlighting the importance of early disease recognition and management.^2^ Melanin serves an important role in the protection of the skin against DNA damage mediated by ultraviolet (UV) rays from the sun; therefore without melanin, skin impacted by vitiligo is exposed to additional susceptibility to sunburns and skin cancers. The burden of vitiligo extends past its physical manifestations; vitiligo patients experience significant reductions in quality of life and increased psychosocial burden.^3^ The complex aetiology creates significant challenges in treatment and management.^4^

Long‐term, robust treatment options for vitiligo are currently limited, highlighting the need for a better understanding of the molecular mechanisms to identify potential drug targets. In this review, we aim to identify and categorise the various pathophysiological mechanisms underlying vitiligo and discuss treatment pathways. By investigating these pathways and identifying potential drug candidates, development of more future precise and clinically relevant therapeutic strategies may ultimately improve the quality of care of vitiligo patients.

## 2 IMMUNE MECHANISMS INVOLVED IN VITILIGO

Vitiligo is an autoimmune skin condition characterised by the loss of melanocytes, responsible for producing skin pigments that offer protection against ultraviolet radiation. The pathogenesis of vitiligo involves a confluence of factors, including autoreactive T‐cells, altered innate and adaptive immunity, genetic predisposition, environmental influences and melanocyte stress.^5^

Central to the most implicated autoimmune mediated processes are cytotoxic T‐cells, which typically identify and eliminate infected or damaged cells. These T‐cells are subjected to a selection process during maturation in the thymus, which eliminates autoreactive variants to prevent self‐tissue harm. However, in vitiligo, autoreactive cytotoxic T‐cells are theorized to develop target melanocytes and release cytokines such as interferon‐γ (IFN‐γ), thereby attracting more cytotoxic T‐cells to the skin. This cycle results in the destruction of melanocytes and the ensuing loss of pigmentation. Treatments targeting IFN‐γ signalling may temporarily ameliorate vitiligo symptoms; however, relapse, particularly upon treatment discontinuation, is common.^6^

Intrinsic abnormalities within melanocytes, coupled with possible environmental triggers causing increased melanocyte stress, can lead to the release of inflammatory signals, potentially triggering autoimmunity.^5^ In addition to the role of adaptive immune system, melanocyte stress also stimulates the innate immune system through the activation of pattern recognition receptors (PRR) in response to danger signals. This mechanism is evidenced by sterile inflammation, where damaged melanocytes produce self‐derived patterns unrelated to pathogens. Under stress, melanocytes generate reactive oxygen species (ROS) and heat shock proteins (HSP), notably HSP70i, which act as damage‐associated molecular patterns in vitiligo. These patterns activate PRR, initiating the inflammatory process. Studies indicate that HSP70i is a critical link between melanocyte stress and the aetiology of vitiligo, primarily through the activation of the innate immune system.^7^

**FIGURE 1:**
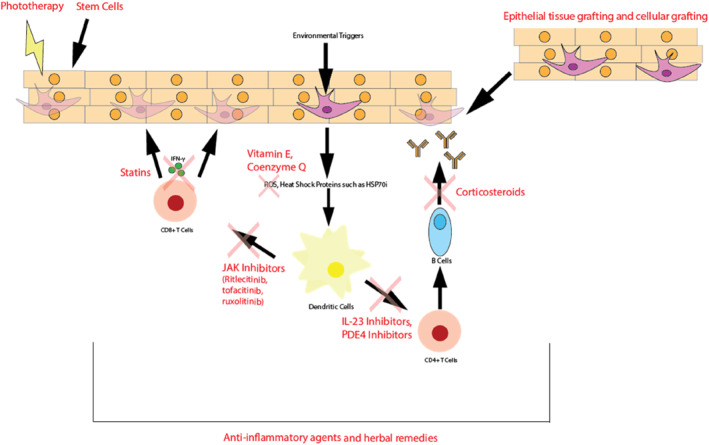
Summary of vitiligo pathophysiology and drug targets of common treatment modalities.

The exploration of druggable pathways include Janus kinase (JAK) inhibitors, which have shown promising results in early trials; tumour necrosis factor (TNF)‐alpha inhibitors and IL17/23 inhibitors, which target specific inflammatory processes; nuclear erythroid 2‐related factor 2/antioxidant response element (Nrf2/ARE), phosphatidylinositol 3‐kinase/protein kinase B (PI3K/AKT) and p38 mitogen‐activated protein kinases (MAPK) pathways, which are integral in cellular signalling and stress responses and the Wnt/beta‐catenin and AhR pathways, crucial in melanocyte biology.^8^, ^9^ Additionally, the IFN‐γ and C‐X‐C motif chemokine ligand 10 axis may play a role in the immunomodulatory aspects of the condition. A summary of the pathophysiology and potential drug targets are illustrated in Figure 1.

## 3 METHODS

A comprehensive search strategy was conducted utilising PubMed, using the Medical Subject Headings terms and keywords ‘vitiligo pathophysiology’, ‘vitiligo interventions’, ‘vitiligo pharmacological interventions’ and ‘vitiligo procedural interventions’. Boolean operators (AND, OR) were used to combine these terms, filtering for English language studies published within the last decade. The review considered various study types, including case reports, case series, cohort studies, meta‐analyses and systematic reviews, addressing vitiligo pathophysiology and interventions. Exclusion criteria included non‐English studies, topics unrelated to vitiligo and articles not discussing a treatment pathway or with insufficient data.

The primary focus of interest encompassed studies exploring vitiligo pathophysiology and evaluating both pharmacological and non‐pharmacological/procedural interventions associated with the condition. Discussion of the pathways implicated and/or identified with clinical utility for vitiligo treatment were emphasised.

### 3.1 Pharmacotherapy

#### 3.1.1 Anti‐inflammatory agents

Inflammatory mediators may play a role in the pathology of downstream autoimmune processes, and thus have been investigated in vitiligo pathogenesis and treatment.^10^ Dysregulation of inflammatory signalling molecules, particularly interleukin (IL) 17, IL‐2, IL‐6, IL‐33 correlates with stages of disease and the degree of the affected area. Low dose of IL's with potent anti‐inflammatory effects (IL‐10, IL‐4), basic fibroblasts growth factor and neuropeptide β‐endorphin improve imbalances in redox homoeostasis.^11^ IL‐17 serum levels are higher in progressive vitiligo compared to stable vitiligo (*p* = 0.014), healthy patients (*p* = 0.002), and in patients with leukotrichia (*p* = 0.04).^12^ CD8+ cytotoxic T‐cells, regulatory T‐cells, and memory T‐cells are implicated in melanocyte destruction. IL‐2 and IL‐15 may play a role in vitiligo development as they activate and promote resident memory T‐cells and signal the JAK/STAT pathways. Janus kinase family proteins are activated by cytokines, and cause melanocyte injury after IFN‐γ is sensed and CYCL9/10 is generated by keratinocytes. However, JAK inhibitor treatment is associated with recurrence after discontinuation of the medication, highlighting disruption of the chemotaxis of cytotoxic cells and non‐removal of memory T‐cells causative of the disease.^13^

The tyrosine kinase expressed in hepatocellular carcinoma (TEC) family has also been implicated in disease evoked by T‐cell‐mediated autoimmunity. Ritlecitinib, an oral JAK3/TEC inhibitor, showed improvements in facial vitiligo scoring index (*p* < 0.001).^14^ Additionally, other inhibitors including tofacitinib (primarily JAK1/3 inhibitor) and ruxolitinib (primarily JAK1/2) inhibitor have shown similar efficacy.^15^, ^16^ Regardless of specific family protein inhibition, inhibitors interfere with the IFN‐γ‐chemokine signalling cascade, preventing further recruitment of activated CDCR3+ CD8+ T‐cells that are responsible for melanocyte apoptosis.

IL‐23 inhibitors, such as ustekinumab and tildrakizumab, have also shown limited efficacy in treating vitiligo. IL‐23 is a central cytokine involved in the pathogenesis of autoimmunity, and it potentiates the differentiation of TH17 cells when in the presence of TGF‐β and IL‐6, thereby inducing and enforcing neutrophil recruitment and inflammatory responses.^17^ IL‐23 levels are elevated in some vitiligo patients.^18^ Use of IL‐23 in regular treatment of vitiligo is limited by the lack of large scale studies evaluating its use in large cohorts of all subtypes of vitiligo.^19^

Phosphodiesterase‐4 (PDE4) inhibition with crisabarole and apremilast may treat vitiligo by inhibiting the degradation of cyclic adenosine monophosphate (cAMP), causing increased intercellular accumulations, thereby preventing T‐helper (Th) and Th‐16 lymphocyte activation and increasing IL‐2/10 expression.^20^ It also decreases pro‐inflammatory mediator secretions including IL‐23, IL‐17, TNF‐α and IFN‐γ.^21^

Corticosteroids have been utilised for localised and general vitiligo treatments and illicit effects by suppressing autoimmune mediated pathways. Theorized mechanisms of action include reductions in complement‐mediated cytotoxicity's by autoantibodies to melanocytes and a decrease in antibody titre to melanocyte surface antigens.^22^

Statins have recently been evaluated for their anti‐inflammatory and immunomodulatory effects on CD8+ mediated inflammatory pathways and inhibition of IFN‐γ production. Statins also upregulate transcription factor nuclear erythroid 2‐regulated factor which activate ROS in melanocytes. Statins may also improve melanogenesis by stimulating the upregulation of α‐melanocyte‐stimulating hormone.^23^ However, not all vitiligo patients utilising statin will receive clinical benefit.^24^

De novo purine synthesis and other DNA precursor (azathioprine, mycophenolate mofetil) inhibitors have been targeted in vitiligo with limited success, and future studies evaluating its clinical efficacy in larger patient cohorts should be conducted.^25^, ^26^ Adverse effects for anti‐inflammatory agents are similar in nature, with heightened infection risk, nasopharyngitis, headache and fever being most common, with limited studies suggesting more selective JAK inhibitor agents potentially having fewer AEs.^27^

#### 3.1.2 Antioxidants/vitamins/herbals

Oxidative stress has been implicated in the underlying pathophysiology of vitiligo. Patients with vitiligo have lower total serum antioxidant status, a higher oxidative stress index and total serum oxidant status in both generalised and localised vitiligo patients.^28^ Additionally, glutathione peroxidase represents a general enzyme family involved in peroxidase reactions, serving to protect cells from oxidative damage by limiting hydroperoxides and reducing hydrogen peroxide to water.^29^ Vitiligo patients are theorized to have low levels of functional glutathione peroxidase levels, which may account for increased susceptibility to oxidative stress leading to disease pathologies. Current lines of evidence are unclear: one meta‐analysis found that vitiligo patients had equivalent levels of glutathione peroxidase compared to healthy controls (standardized mean difference −0.47, 95% CI: −1.03 to 0.08). However, glutathione peroxidase levels in serum/plasma were low in stable/active vitiligo patients (SMD = −2.01, 95% CI −3.52 to −0.49, *p* = 0.009). Further subgroup analyses found that patients who were Asian and who had segmental vitiligo had lower glutathione activity (*p* = 0.001 and 0.012).^30^

Antioxidant pathways are currently being investigated for potential clinical utility in vitiligo. The NRf2/ARE pathway may upregulate antioxidant gene expression and the PI3K/AKT may regulate melanocyte proliferation and maturation. Additionally, the Wnt/B‐catenin pathway may stimulate repigmentation through melanocyte stem cells, and AhR may aid in repairing mitochondrial oxidative damage. The P38 MAPK may reduce oxidative stress to melanocytes and promote melanogenesis and antioxidant activity.^8^ The most common antioxidants used for treatment include pseudo catalase, vitamin E, coenzyme Q, ginkgo biloba, *Polypodium leucotomos* and zinc, yet clinical studies evaluating their clinical efficacy is limited.^8^, ^31^ Although 90.7% of dermatologists agree with the oxidative stress theory of vitiligo, only 56.3% regularly encourage antioxidant use for treating vitiligo, and only 11.8% actively treat patients with it. Lack of knowledge regarding antioxidant use was correlated with lower frequency of use (*p* < 0.001).^32^

Similarly, vitamin and mineral deficiencies may be significant in vitiligo's development, impacting immune responses and skin health. In a cohort of Iranian vitiligo patients, serum copper and zinc levels were low compared to healthy controls (*p* = 0.01), potentially linking altered copper and zinc levels to the development of vitiligo.^33^ Additionally, lower 25‐hydroxyvitamin D levels were observed in children with vitiligo, suggesting a potential association between vitamin D and the onset of vitiligo in this age group.^34^

An evaluation of herbal remedies has helped elucidate some mechanisms of vitiligo pathogenesis and paved the way for potential drug targets. The Qubaibabuqi formula, which contains over 83 therapeutic proteins, is involved with the melanogenesis, serotonergic, calcium signalling, chemokine, PI3K‐AKT and toll‐like receptor pathways. Some vital active ingredients including butin, psoralen, kaempferol and cholesterol play a role in immunomodulation, neuromodulation and inhibiting keratinocyte apoptosis.^35^ Another herbal medication containing *Psoralea corylifolia* (*PC*) from seed powder was formulated into a hydrophilic ointment and evaluated in 20 vitiligo patients. The ointment was effective in increasing post‐treatment levels of pigmentation in small circular white lesions (*p* ≤ 0.05).^36^ PC contains many constituents, including psoralen, isopsoralen, falvones, bavachinin, bakuchiol, etc, that have antioxidant, antibacterial and immunomodulatory activities.^37^ Similarly, an herbo‐mineral capsule (ALG‐06) consisting of two minerals (red ochre and purified sulphur) and the seeds of three plants (*Psoralea corylifolia* L., *Punica granatum* L., *Trigonella foenum‐graecum* L.) was developed in 500 mg capsules, and it was found to be stable (however not yet tested in vivo).^38^

Piperine, an alkaloid of black pepper (*Piper nigrum* [PN]), stimulates the proliferation of melanocytes and increases the number and length of cell dendrites. Furthermore, growth has been found to be inhibited by RO‐31‐8220, a selective protein kinase C (PKC) inhibitor, which suggests that PKC signalling is involved in piperine activity.^39^ The efficacy of PN extract and pure piperine have been compared in treating vitiligo. Treatment with PN extract ointment led to faster results and pigmentation islands while treatment with piperine ointment led to diffuse pigmentation. This finding suggests that additional compounds may be present in the PN extract that are involved in the pigmentation process. When the PN extract ointment was associated with travoprost solution, pigmentation occurred faster and both pigmentation islands and diffuse pigmentation were observed.^40^ In one clinical trial, greater repigmentation was seen in patients treated with topical piperine combined with NB‐UVB than in patients who were treated with only NB‐UVB (repigmentation scores at 3 months of 53.6 and 16.3, respectively, *p* < 0.001). Temporary AEs of irritation and redness of the skin were observed in patients treated with piperine and were treated with zinc oxide ointment.^41^

Vehicle delivery systems for piperine have also been studied, with ethanol (*E*th) achieving the highest piperine solubility (48.6 mg/mL) and flux (40.8 μg/cm^2^h) in a study that also tested propylene glycol (PG), polyethylene glycol, and oleic acid (OA). Among combination systems, the 75:25 Eth‐OA system achieved the highest piperine flux (59.3 μg/cm^2^h). The solubility of piperine was significantly increased in the PG‐OA (50:50), Eth‐OA (25:75) and Eth‐OA (50:50) systems (20.1, 43.9, and 43.1 mg/mL respectively).^42^ A topical piperine nano emulsion has been developed and has shown a statistically significant increase in tyrosinase activity (32.77% ± 9.09%) and melanogenesis activity (34.90% ± 0.73%) in melanoma B16 cells at a 5 mg/mL dose.^43^

### 3.2 Phototherapy

Phototherapy such as narrow band ultraviolet B (NB‐UVB) and psoralens with ultraviolet A (PUVA), is another treatment option for vitiligo. NB‐UVB specifically is utilised in vitiligo treatment due to its ability to stimulate melanocyte activity and proliferation. Affected areas of vitiligo may respond to the UVB radiation by potentially reactivating dormant melanocyte precursors or promoting the migration of melanocytes from hair follicles into the depigmented skin. PUVA on the other hand, is a procedure combining a photosensitising agent with UVA exposure and is used for extensive or resistant cases but carries a higher risk profile, including potential skin ageing and cancer risks.

In a meta‐analysis including 35 studies and 1428 different patients, patients treated with NB‐UVB treatment had at least mild response in 62.1% at 3 months, 74.2% at 6 months and 75.0% at 12 months.^44^ A marked response occurred in 13.0% at 3 months, 19.2% at 6 months and 35.7% at 12 months. In terms of PUVA therapy, a least mild response occurred in 51.4% at 6 months and 61.6% at 12 months.

Phototherapy, particularly NB‐UVB, has been extensively studied for its efficacy and safety profile. Treatment with NB‐UVB demonstrate significant repigmentation, particularly in cases of non‐segmental vitiligo.^45^, ^46^ While the treatment regimen typically involves multiple weekly sessions over several month, the AEs such as erythema or minor itching are generally well‐tolerated.^47^, ^48^ A comparative prospective clinical trial study compared oral PUVA with PUVA solution, found oral PUVA more efficacious, and both treatments reported phototoxicity effects such as burning and itching.^49^

### 3.3 Procedural modalities

Various dermato‐surgical techniques and modalities that utilise many procedures involving dermabrasions, lasers, micro‐needling and suction blistering have been studied for vitiligo treatment.^50^ Although procedural treatments are generally not considered as first‐line treatments, vitiligo procedures have been increasingly studied in clinical trials.^51^ These techniques are most effective in segmental vitiligo due to increased disease stability. Both tissue epithelial grafting and cellular grafting are invasive procedures aimed at restoring melanocyte function at central depigmentation areas for pigment restoration (Figure 1). Potential complications include an invasive procedure, coinciding with pain, oedema, scarring and potential delays in healing.

#### 3.3.1 Epithelial tissue grafting

Tissue grafting is based on the principle of taking a fragment of stable unaffected skin with proper pigmentation and transferring it to vitiligo‐affected areas. The method to harvest the donor cells is varied, with dermabraders, fractional ablative lasers and micro needling being used. Different surgical methods of tissue grafting include mini punch, split‐thickness, epidermal curettage, smash, flip‐top and hair follicle grafting.^50^ Suction blister epidermal grafting (SBEG) is a procedure that allows repigmentation by creating a subepidermal bulla from the donor site using a vacuum application followed by surgical removal of the roof and transplantation to the recipient site. Negative pressure is often generated at the donor site and a heating source is utilised to shorten blistering time and promote graft uptake.^52^ Donor site cells are generally non‐UV‐exposed surfaces such as the thigh, buttocks and glutaeal region.^53^ SBEG is highly effective, with repigmentation occurring in as many as 70% of treated cases.^54^ Another technique, automated blister epidermal micrograft (ABEM), is a less painful alternative with reduced donor site trauma. Similar to SBEG, ABEM utilises a negative pressure of −400 to −500 mmHg at a temperature of around 40°C and produces suction microdomes to harvest epidermal micrografts. Tacrolimus 0.1% is regularly applied after the grafting procedure with possible ultraviolet B (UVB) phototherapy to improve graft success.^52^ SBEG has higher repigmentation and vitiligo areas scoring index (both *p* < 0.001) and is less expensive; however, ABEM is easier to learn and can supply a greater transplanting zone with possibly less recovery times.

Regardless of the treatment procedure specifics, the principle of tissue grafting remains the same: extract epithelium with functional melanocytes and transplant them in areas of affected vitiligo and allow for tissue healing. The repigmentation resulted from activation of melanocytes located in the donor sites, which migrate horizontally towards the affected skin due to intercellular spaces coupled with decreases in E‐cadherin reactivity and up regulation of pro‐melanogenic signals.^55^ Vitiligo melanocytes are known to have intrinsic deficits limiting their survivability and stability making them susceptible to internal and external auto‐inflammatory mechanisms, attributed to mosaicism of body melanocytes.^56^, ^57^ Epithelial grafting allows for new melanocyte populations that do not possess the same genetic vulnerability to restore pigment to affected areas that are less susceptible to reactivation.^58^, ^59^

#### 3.3.2 Cellular grafts

Cellular grafting therapies have been utilised as a strategy for wound repair through culture cells that are processed and transformed to serve as grafting material. Types of cellular grafts used include cultured melanocyte and epidermal grafts, non‐cultured epidermal melanocyte suspensions and non‐cultured follicular root sheath suspension. Autologous cultured epithelial cells have been used in vitro to regenerate epithelial cells with extensive proliferative self‐renewal.^60^ Creation of large quantities of cultured epidermal grafts with intact, functional melanocytes has a high efficacy of 77% rate of repigmentation at 12–36 months after follow up, with lower repigmentation in extremities (8%) and periorificial sites (35%).^61^ Recent techniques have shown that epithelial cells transplantation is a relatively safe and effective therapy for facial segmental stable vitiligo patients.^60^ Cultured epithelial cells can be inoculated in pure cell suspension or co‐cultured with other epithelial cells (melanocytes + keratinocytes). Cultured epidermal sheets may lead to repigmentation by creating a ‘lake’ of cultured melanocytes to the transplanted area as opposed to the ‘horizontal migration’ consistent with other epithelial cell grafting techniques.^61^

Advantages of this method include creating many new cells using small donor material allowing for transplanting a large body surface area, and the absence of scar formation. However, quality control of the developed culture system is an important limitation, including appropriate melanocyte–keratinocyte ratio and ensuring no depletion of epidermal stem cells.^61^

Another regenerative medicine technique that has been evaluated in vitro is the use of adipose tissue secretome as a cell free therapy. Adipose tissue extracellular fraction can improve cell proliferation and survival by counteracting oxidative stress by enhancing function of innate antioxidant properties. Initial in vitro studies have shown enhanced cellular protective effects through Wnt‐secreted factors promoting glycogen synthase inactivity.^62^

### 3.4 Genetic polymorphisms

Autoimmune conditions typically develop due to an environmental exposure in a genetically susceptible individual. Therefore, genetic polymorphisms play an important role in vitiligo: shaping susceptibility, disease progression and response to treatments and offering insights into its multifactorial aetiology and potential for targeted therapeutic approaches.

Certain pigmentation mechanisms emphasise melanocytes' key role under melanocyte‐specific microphthalmia‐associated transcription factor (m‐MITF) regulation. UV radiation influences melanogenesis via the α‐melanocyte‐stimulating hormone (α‐MSH) and melanocortin 1 receptor (MC1R), with mutations potentially being implicated in vitiligo pathogenesis.^63^ Catalase (CAT) gene variations were studied in Sicilian vitiligo patients, revealing an equal distribution of genotypes compared to Northern Europe and the Mediterranean. Specifically, only the CAT‐89T/T polymorphic variant showed higher frequency in vitiligo cases, suggesting its potential relevance.^64^ Targeted cancer therapies, including epidermal growth factor receptor and breakpoint cluster region‐Abelson inhibitors have also been associated with reduced pigmentation in treatment‐refractory vitiligo.^65^

Noncoding RNAs, notably microRNAs (miRNAs), may play a role in vitiligo susceptibility, regulating processes such as melanin metabolism and cell survival. Studies have investigated specific variants, such as miRNA‐211 and its impact on cellular metabolism. Reduced miRNA‐211 expression in vitiligo, along with altered gene expression patterns and impaired mitochondrial function, suggest its involvement in vitiligo pathogenesis, offering potential biomarkers and therapeutic avenues.^66^, ^67^

Toll‐like receptors (TLRs) have also been implicated in vitiligo. TLR2 and TLR4 gene polymorphisms were discovered in a cohort of 100 Turkish vitiligo patients, specifically elevations in the TLR2 Arg753Gln and TLR4 Asp299Gly variants.^68^ Other key pathways have been studied in relation to vitiligo, including Wnt/β‐catenin, melanogenesis and NOD‐like receptor signalling, with the Wnt/β‐catenin pathway being most closely associated with pigmentation.^69^

Angiotensin converting enzyme (ACE) have traditionally been thought to primarily regulate vascular physiology, blood pressure and more recently have been implicated in inflammatory processes such as vitiligo. Serum levels of ACE, IL‐6 and nitrite in vitiligo patients were significantly elevated (*p* = 0.007).^70^

Koebner phenomenon (KP) triggers new dermatosis at sites of mechanical trauma and is often linked to chronic friction in vitiligo. In one study of 202 vitiligo patients, KP was observed in 130 individuals who were associated with an older mean age at onset (23.9 years vs. 19.3 years; *p* = 0.02), larger skin involvement (4.6% vs. 1.5%; *p* = 0.001) and a higher likelihood of receiving systemic steroids (55 patients vs. 9; *p* = 0.01), suggesting a potential association between KP subtypes and genetic variations influencing disease characteristics.^71^ Another study of 402 vitiligo patients found itch in 20.2% of cases, most prevalent in focal vitiligo (29.4%).^72^ Itch preceded lesions by 3 days in 48.1% of patients. Those with itch and KP type IIb frequently had active vitiligo, suggesting a potential link between itch, especially in conjunction with KP type IIb and active disease states.

### 3.5 Stem cells

Stem cells have a multifactorial role in vitiligo pathogenesis, often studied in relation to melanocyte loss. Understanding the niche and behaviour of melanocytes derived from stem cells (MelSCs), which regulate melanocyte maintenance and regeneration, holds promise for treating pigmentation disorders.^73^ While murine models have offered insights, the study of human MelSCs and their isolation remains less explored. Identifying and culturing human MelSCs can pave the way for novel therapies addressing pigmentary disorders, potentially offering new treatment avenues in the future.

Combined therapy of NB‐UVB phototherapy and adipose‐derived stem cell transplantation was examined for vitiligo in a mouse model, focusing on endoplasmic reticulum stress and calcium homoeostasis. This treatment approach showed promise in restoring redox balance and cellular calcium levels, reducing oxidative stress markers and reversing ROS levels which are diagnostic markers of vitiligo, indicating a potential reduction in endoplasmic reticulum (ER)‐mitochondrial stress and Ca^2+^ overload. This approach addresses the theory behind oxidative stress and Ca^2+^ imbalances and their association with vitiligo.^74^

Adipose tissue extracellular fraction (AT‐Ex) promotes skin repair processes through increased dermal and epidermal cell proliferation along with enhanced fibroblast migration.^75^ AT‐Ex also showed positive effects against oxidative stress damage and prevented fibroblast senescence, suggesting its potential in improving wound healing and tissue repair in vitiligo.

**FIGURE 2:**
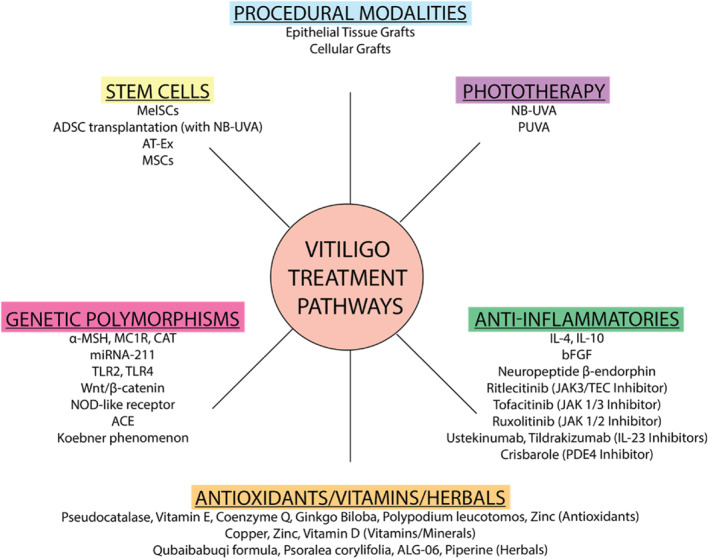
Summary of vitiligo treatment pathways and drug targets.

Other studies report non‐enzymatic protocols to harvest regenerative cells from adipose tissue, utilising centrifugation of the infranatant fraction of lipoaspirate as enrichment steps.^76^ These cells, meeting criteria for mesenchymal stem cells, offer potential for treating vitiligo, as well as burns and chronic wounds. A summary of the treatment options, clinical evidence, advantages and disadvantages for various treatment options is summarised in Table 1 and Figure 2.

**TABLE 1:** Summary of key vitiligo treatment approaches.

Treatment modality	Subclass	Key features	Clinical evidence	Advantages	Disadvantages
Procedural modalities	Epithelial tissue grafting	Transfer of stable pigmented skin to vitiligo‐affected areas. Various methods (dermabraders, lasers and micro needling), donor site selection (thigh, buttocks)	High repigmentation rates (up to 70%)^54^	Effective for stable vitiligo	Invasive, risk of pain, scarring, longer healing time
	Suction blister epidermal grafting (SBEG)	Creation of subepidermal bulla for grafting. Negative pressure application, donor site (non‐UV‐exposed areas)	High repigmentation rates (up to 70%)^54^	Less expensive than alternatives	Potential for blister‐related complications
	Automated nlister epidermal micrograft (ABEM)	Automated suction microdomes for epidermal micrografts. Negative pressure application, reduced donor site trauma	Similar efficacy to SBEG, easier to learn^52^	Larger transplant area, shorter recovery	Limited by equipment availability
Phototherapy	Narrow band ultraviolet B (NB‐UVB)	UVB therapy to stimulate melanocyte. Multiple sessions over weeks/months	Significant repigmentation in non‐segmental vitiligo^45^, ^46^	Well‐tolerated side effects (erythema, itching)	Requires frequent clinic visits
	Psoralens with ultraviolet A (PUVA)	UVA therapy with photosensitising agent. Combination therapy for extensive/resistant cases	Effective but higher risk profile (skin ageing, cancer risks)^49^	Treats larger areas	Risk of phototoxicity
Pharmacotherapy	JAK inhibitors	Inhibits JAK/STAT pathway to prevent melanocyte destruction. Examples include tofacitinib, ruxolitinib and ritlecitinib	Improvements in vitiligo scoring indices^15^, ^16^	Targeted therapy prevents cytotoxic cell recruitment	Recurrence after discontinuation
	IL‐23 inhibitors	Targets IL‐23 involved in autoimmune response. Examples include ustekinumab, tildrakizumab	Limited efficacy data in vitiligo^19^	Potential for systemic use	Limited large‐scale studies
Anti‐inflammatory agents	Corticosteroids	Suppresses autoimmune pathways. Localised treatment option	Effective for localised vitiligo^22^	Potential for systemic side effects	Long‐term use limitations
	PDE4 inhibitors	Inhibits PDE4 to modulate inflammatory responses. Examples include crisaborole and apremilast	Reduction in pro‐inflammatory mediators^21^	Topical application, no systemic side effects	Limited efficacy in severe cases
Antioxidants/vitamins/herbals	Vitamin E, coenzyme Q	Antioxidant therapy to reduce oxidative stress. Various antioxidants (vitamin E, coenzyme Q)	Limited clinical evidence^31^, ^32^	Low risk of adverse effects	Variable efficacy across patients
	Herbal remedies	Plant‐based therapies with immunomodulatory effects. Examples include *Psoralea corylifolia* and *Piper nigrum* extract	Improved pigmentation outcomes^36^	Natural compounds, potential for adjunct therapy	Lack of standardized formulations
Genetic polymorphisms	Candidate genes	Genes influencing vitiligo susceptibility and treatment response. Examples include CAT, MC1R and TLRs^63^	Insights into disease mechanisms	Potential for targeted therapies	Complex genetic interactions are not yet fully characterised

## 4 CONCLUSION

Vitiligo is an autoimmune mediated disease characterised by functional loss of melanocytes. Current therapies are aimed at drug pathways to restore melanocyte function and restore pigment in the affected areas. Treatment modalities for vitiligo involve procedural tissue and cellular grafting, as well as phototherapy to replace non‐functional melanocytes with functional cells and stimulate melanocytes. Regulation of downstream pro‐inflammatory pathways, including the JAK‐stat pathway, ILs and PDE4, have been targeted to prevent CD8+ T‐cell mediated melanocyte destruction. Genetic targets, including a‐MSH, TLRs and Wnt/B‐catenin, have also been investigated as potential drug targets and antioxidants, including vitamin D, E, copper and piperine, have been evaluated as potential agents in limited studies. Stem cells aimed at restoring normal melanocyte function represent a promising modality for future research. Overall, current treatment options for vitiligo are limited, and a deeper understanding in the implicated pathways may drive future drug development targets and guide a multimodal treatment approach.

## CONFLICT OF INTEREST STATEMENT

Dr. Babar Rao is a speaker for Incyte. All other authors have no disclosures.

## AUTHOR CONTRIBUTIONS

**Gaurav N. Pathak**: Conceptualization (lead); investigation (equal); resources (equal); writing—original draft (lead); writing—review and editing (equal). **Isabella J. Tan**: Conceptualisation (equal); investigation (equal); writing—original draft (equal); writing—review and editing (equal). **Ge Bai**: Conceptualization (equal); investigation (equal); methodology (equal); writing—original draft (equal); writing—review and editing (equal). **Jimmy Dhillon**: Conceptualization (supporting); investigation (supporting); methodology (supporting); resources (supporting); writing—original draft (supporting); writing—review and editing (equal). **Babar K. Rao**: Supervision (supporting); validation (equal); writing—original draft (supporting); writing—review and editing (lead).

## ETHICS STATEMENT

Not applicable.

## PATIENT CONSENT

Not applicable.

## Data Availability

Data sharing is not applicable to this article as no new data were created or analysed in this study.
